# Management of Class III Malocclusion and Maxillary Transverse Deficiency with Microimplant-Assisted Rapid Palatal Expansion (MARPE): A Case Report

**DOI:** 10.3390/medicina58081052

**Published:** 2022-08-04

**Authors:** Sin-Ni Shih, Kwok-Hing Ho, Chih-Wei Wang, Kai-Long Wang, Shun-Chu Hsieh, Heng-Ming Chang

**Affiliations:** Department of Orthodontics, Chang Bing Show Chwan Memorial Hospital, Changhua 505, Taiwan

**Keywords:** microimplant-assisted rapid palatal expansion, class III malocclusion, maxillary skeletal expander, maxillary transverse discrepancy

## Abstract

Microimplant-assisted rapid palatal expansion (MARPE) has been demonstrated successfully in maxillary expansion in late adolescence and adulthood. The maxillary advancement accompanied by expansion is frequently anticipated, which is beneficial for the treatment of class III malocclusion. Airway volume increase can also be noted in some cases from the measurement of cone beam computerized tomography (CBCT) after expansion. The objective of this case report is to demonstrate the feasibility of applying MARPE on late adolescence patients with maxillary transverse deficiency and to present the changes in transverse and anteroposterior dimensions as well as the volume increase in velopharyngeal airway after MARPE. A 15-year-old female presented class III skeletal pattern. She had maxillary transverse deficiency with moderate crowding and posterior/anterior crossbites. Maxillary Skeletal Expander (MSE; Biomaterials Korea Inc.) type-2 was used as a MARPE device in this case. After four weeks of maxillary expansion, a significant amount of expansion was achieved and the anterior crossbite was spontaneously corrected. Fixed appliance treatment was commenced four weeks after MARPE with 0.022-slot preadjusted brackets (MBT prescription). Temporary anchorage devices (TADs) were placed over the mandibular buccal shelves for posterior teeth distalization and crowding relief. After 25 months of treatment, the facial profile was improved with maxillary advancement (SNA: 83° to 83.5°) and mandibular backward rotation (SNB: 83° to 82°; SN-MP: 34.5° to 35°). In this case, MARPE not only engenders significant transverse correction but also aids in anteroposterior change. The treatment effects of maxillary advancement and mandibular backward rotation can lead to a more esthetic profile in skeletal class III cases.

## 1. Introduction

For class III malocclusion, patients frequently present maxillary retrusion, mandibular protrusion, or a combination of both [1]. These problems also result in maxillary constriction in transverse dimension, accompanying anterior and posterior crossbites. Thus, treatment of class III malocclusion should be addressed not only on the anteroposterior relationship but also on the transverse dimension, since they are both out of balance [2].

Conventionally, rapid palatal expansion (RPE) devices, e.g., Hyrax or Haas expanders, are used to correct narrow maxillary arch in growing patients by a combination of orthopedic and dental expansion. By opening the midpalatal suture, RPE devices are effective in producing transverse skeletal effects on the maxilla in growing children. After expansion, the midpalatal suture was recognizable, and the expansion was believed to be stable [3]. Bizzarro et al. [4] found that subjects with buccally displaced canines (BDC) have significantly reduced maxillary intercanine widths. With a thorough examination, they suggested timely intervention, e.g., RPE, may allow prevention of maxillary canine impaction in patients with maxillary deficiency [4]. However, undesirable side effects including buccal tipping of anchored teeth, periodontal membrane compression, buccal root resorption, alveolar bone bending, fenestration of the buccal cortex, unfavorable periodontal consequences, and a lack of long-term stability are inevitable [5]. Moreover, less effective expansion and limited skeletal effect have also been observed clinically in nongrowing patients due to maturation of the midpalatal suture and adjacent articulations [6].

Because of more complications and limited skeletal effects of RPE in skeletally mature patients, surgical procedures have been recommended to overcome the resistance of the progressively fused and matured sutures [7]. Surgical-assisted rapid palatal expansion (SARPE) is recommended to be performed in patients with complete skeletal maturity and closed cranial sutures, whose transverse maxillary deficiencies are greater than 5 mm [8]. However, some complications were reported, e.g., significant hemorrhage, injury to the branches of the maxillary nerve, pain, sinus infection, alar base flaring, and relapse, which may affect patient acceptability [7].

The increasing demand for nonsurgical maxillary expansion in late adolescents and adults stimulated the development of microimplant-assisted rapid palatal expansion (MARPE) by Lee et al. [9] and Moon et al. [10]. It was designed to maximize skeletal effects and minimize the dentoalveolar effects of expansion by incorporating microimplants into the palate, delivering the expansion force directly to the basal bone of the maxilla [11]. In a recent systematic review, MARPE showed a high success rate of 92.5% on transverse maxillary expansion, ranging from 80.65% to 100% [11]. MARPE seems to provide an optimistic alternative in late adolescents and adults if the patient is contraindicated for SARPE or multiple-piece orthognathic surgery [9,12].

Liao et al. [13] urged that the skeletal effects of maxillary advancement and mandibular backward rotation associated with MARPE can provide a positive correction in class III malocclusion. Several studies also showed similar trends with an increase in SNA after MARPE [14,15]. This effect is similar to the conventional RPE, which has been mentioned in the earlier RPE studies [16,17,18].

This case report aims to demonstrate the feasibility of applying MARPE in late adolescence with class III malocclusion and maxillary transverse deficiency.

## 2. Case Report

### 2.1. Diagnosis and Aetiology

A 15-year-old female presented class III skeletal pattern with average Frankfort-mandibular plane angle along with anterior/posterior crossbites and dental crowding. The patient was generally fit and well, and no relevant medical or allergy history was reported.

**Figure 1:**
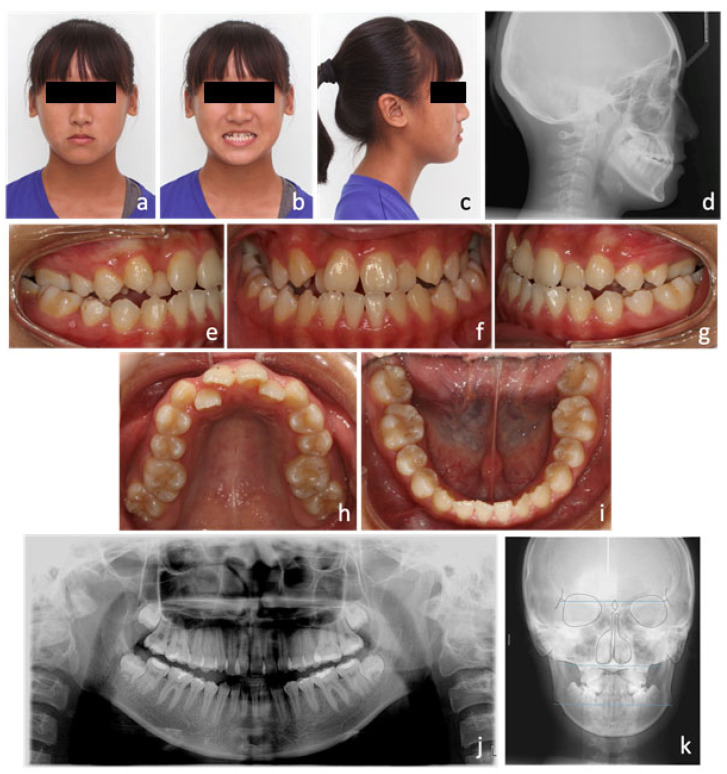
Initial photos and radiographs. (**a**) Extra-oral photos showed flat midface and mild facial asymmetry with chin deviation to the right. (**b**) Vertical facial height proportion was balanced but the upper incisor show was minimal. (**c**) Lateral profile showed a mild concave profile. (**d**) Skeletal pattern was class III with an average Frankfort-mandibular plane angle from the cephalometric radiograph. (**e**–**i**) Intra-oral photos showed moderate crowding (5.4 mm deficit) in the upper arch, mild crowding (1.9 mm deficit) in the lower arch, and bilateral crossbites in the buccal segments. (**j**) The initial orthopantographic (OPG) radiograph. (**k**) Maxillomandibular transverse discrepancy was 35.5 mm measured from Ricketts Rocky Mountain analysis.

An extra-oral examination revealed a mild concave profile with the upper lip retrusive to the E-line (−3.5 mm). The patient’s midface was flat and lacked fullness over the malar region. Vertical facial proportion was balanced but the upper incisor show was minimal. From the frontal view, mild facial asymmetry was noted with chin deviation to the right (Figure 1a–c).

An intra-oral examination demonstrated moderate crowding (5.4 mm space deficiency) in the upper arch and mild crowding (1.9 mm space deficiency) in the lower arch. The maxilla was constricted with both upper lateral incisors palatally displaced. In occlusion, canine and molar were both in class III relationships (Angle classification) on both sides. Incisor relationship was also class III (British Standard Institute, London, United Kingdom, 1983) with negative overjet (−1 mm) and reduced overbite (0 mm). Upper and lower dental midlines were both deviated to the right for 2 mm and 1 mm, respectively. Bilateral crossbites were noted in the buccal segments (Figure 1e–i).

The orthopantographic (OPG) radiograph found no remarkable caries or any pathological condition, and the periodontal status was good (Figure 1j). The cephalometric analysis confirmed the diagnosis of skeletal class III relationship with average Frankfort-mandibular plane angle (SNA: 83°, SNB: 83°, ANB: 0°, and SN-MP: 34.5°) (Figure 1d). The lower labial segment was slightly retroclined (L1-MP: 87°) due to dental compensation. Maxillomandibular transverse discrepancy was 35.5 mm measured from Ricketts Rocky Mountain analysis [19] (Figure 1k; Table 1).

### 2.2. Treatment Alternatives

Betts et al. [20] introduced a maxillomandibular transverse differential index, which was defined as the difference between actual transverse difference of patient and expected transverse difference measured from Rocky Mountain analysis. When this index is larger than 5 mm, orthopedic correction, such as RPE, SARPE, or orthognathic surgery should be considered for the correction of transverse discrepancy (Figure 1k; Table 1).

However, in adolescence, it would be too late to apply conventional RPE for transverse correction since the sutures adjacent to the maxilla halves have begun to fuse and ossify [21]. On the other hand, SARPE or orthognathic surgery would be too early for this age group of patients. Thus, MARPE seems to provide a practical alternative for the correction of maxillary transverse deficiency in adolescence or later.

**Figure 2:**
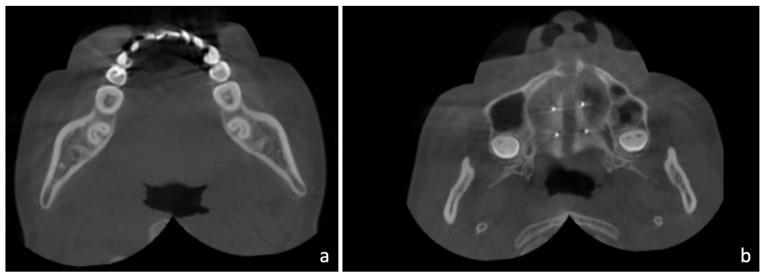
Axial sections of the cone beam computerized tomography (CBCT). (**a**) Distal roots of second molars were close to the lingual cortex of the alveolar process, which means the spaces for posterior distalization are minimal. (**b**) Disengagement of the pyramidal process from the pterygoid notch was noted in the left pterygopalatine suture. The opening is present between the pyramidal process and the lateral pterygoid plate.

When trying to apply a non-extraction method in class III malocclusion, it is important to learn the posterior anatomic limit of mandible, i.e., lingual cortex behind lower second molar, and to see if space is enough for distalization [22,23]. In this case, we examined available spaces behind the lower second molars and found the spaces were very minimal on both sides (Figure 2a). In addition, the maxilla was slightly retrusive, so upper arch advancement instead of lower arch distalization should be more emphasized in this case. Fortunately, only mild crowding (1.9 mm space deficiency) was presented in the lower arch. Therefore, we aimed to treat this case by maxillary advancement, as an adjunctive result of MARPE, and limited lower arch distalization for crowding relief with temporary anchorage devices (TADs) over the buccal shelves. A more esthetic profile was anticipated after the treatment.

### 2.3. Treatment Progress

A maxillary skeletal expander (MSE; Biomaterials Korea Inc., Seoul, South Korea) type-2 was used as the MARPE device in this case. It was straddled along the midpalatal suture and placed at the level of upper first molars. Four microimplants (1.8 mm in diameter; 11 mm in length; Biomaterials Korea Inc., Seoul, South Korea) were inserted and bicortical penetration of the miniscrews was recommended, as it was believed this was fundamental to support anchorage during expansion and to overcome the resistance of the maxillary bone [24]. After 2 weeks of healing, maxillary expansion was commenced with three turns per day for 3 weeks until posterior crossbites was corrected. Midpalatal suture was successfully opened with minimal buccal tipping of the posterior teeth. After 4 weeks of retention, fixed appliance treatment was commenced with 0.022-slot preadjusted brackets (MBT prescription; 3M, Maplewood, Minnesota, USA). After five months of leveling and alignment, TADs were placed over the mandibular buccal shelves on both sides for molar distalization and crowding relief. During the finishing stage, some brackets were repositioned for root parallelism, and interarch elastics were used for occlusion settling. After 25 months of treatment, brackets were de-bonded and the MSE device was removed. Vacuum-formed clear retainers were prescribed for further retention.

### 2.4. Treatment Results

**Figure 3:**
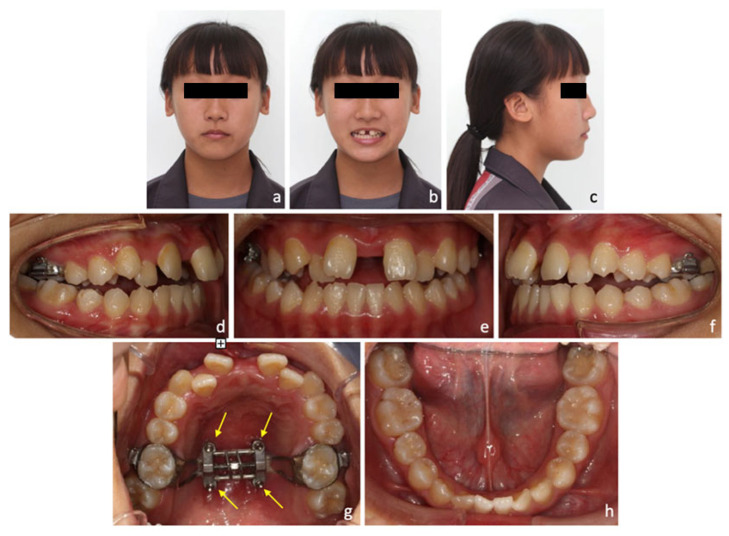
Post-expansion photos. (**a**–**c**) Extra-oral photos showed median diastema. (**d**–**h**) Intra-oral photos showed correction of anterior crossbite and presence of anterior open bite. (**g**) MSE device placed in the upper arch with yellow arrows showing the position of four microimplants.

After 4 weeks of maxillary expansion, anterior crossbite was spontaneously corrected as a result of maxillary advancement (SNA: 83° to 84°), upper incisor proclination (U1-SN: 108° to 112°), and mandibular backward rotation (SNB: 83° to 82°) after MARPE. Median diastema was noticed from extra-oral and intra-oral photos right after expansion (Figure 3).

**Figure 4:**
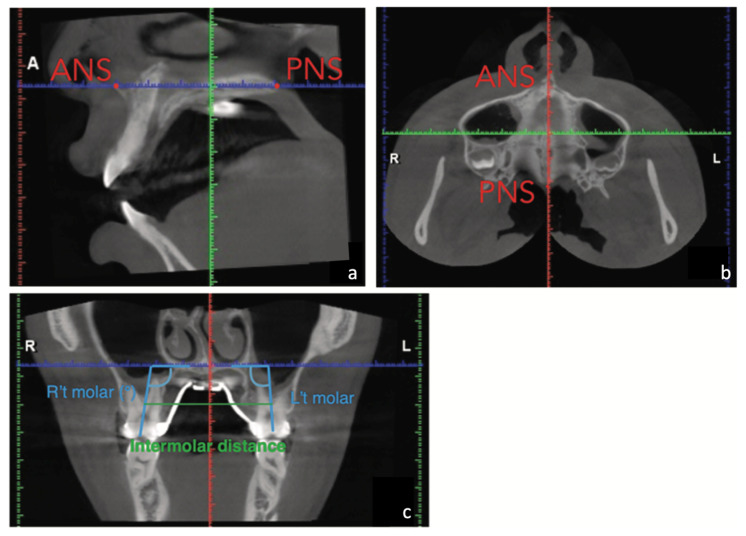
Head orientation of the cone beam computerized tomography (CBCT) images. (**a**) The sagittal plane was determined by the points of anterior nasal spine (ANS), posterior nasal spine (PNS), and the most anterior point of the vomer (V). (**b**) The axial plane was set perpendicular to the sagittal plane and parallel to the palatal plane (ANS-PNS). (**c**) The coronal plane was perpendicular to these two reference planes and passed through the level of the trifurcations of maxillary first molars.

A significant amount of expansion was achieved. The dental and skeletal changes after maxillary expansion were measured on the CBCT. The head orientation of the CBCT images was determined by the method of Liao et al. [13]. The sagittal plane of the CBCT images was determined by the points of anterior nasal spine (ANS), posterior nasal spine (PNS), and the most anterior point of the vomer (V). The axial plane was set perpendicular to the sagittal plane and parallel to the palatal plane (ANS-PNS). The coronal plane was perpendicular to these two reference planes and passed through the level of the trifurcations of maxillary first molars (Figure 4).

**Figure 5:**
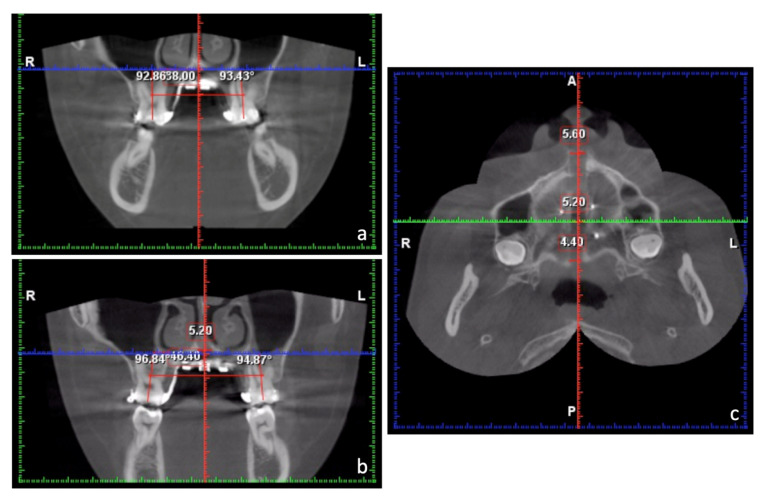
Measurements on the cone beam computerized tomography (CBCT). (**a**) Intermolar distance was defined as the distance between the trifurcations of right and left maxillary first molars. Molar angulation was measured between the axial plane and the long axis of the maxillary first molar. (**b**) Pre-expansion measurements: intermolar distance (38 mm) and molar angulation (92.86° on the right; 93.43° on the left). (**c**) Post-expansion measurements: intermolar distance (46.4 mm), intermolar expansion: (8.4 mm); molar angulation (94.84° on the right; 94.87° on the left), angulation changes (1.98° on the right; 0.44° on the left). In the axial plane, the amounts of skeletal expansion on palatal plane at anterior nasal spine (ANS), first molar and posterior nasal spine (PNS) were 5.6 mm, 5.2 mm and 4.4 mm, respectively. A fairly parallel expansion pattern was demonstrated in the antero-posterior (AP) direction (PNS/ANS: 78.6%).

On the coronal plane, 8.4 mm of intermolar expansion was noted in this case by measuring the increase of intermolar distance (38 mm to 46.4 mm), which was defined as the distance between trifurcations of right and left maxillary first molars. Molar angulation changes, which were defined as the long axis change of the first molar on the coronal plane, were small (2° on the right side; 1.4° on the left side), and these demonstrated minimal dentoalveolar buccal tipping after MARPE. The amount of skeletal expansion at the palatal level on the coronal plane of trifurcations of first molars was 5.2 mm, which accounted for 62% of intermolar expansion (Figure 5a,b). On the axial plane at palatal level, 5.6 mm and 4.4 mm expansions were measured at the ANS and PNS, respectively, which demonstrated a fairly parallel expansion pattern in the antero-posterior (AP) direction (PNS/ANS: 78.6%) (Figure 5c).

**Figure 6:**
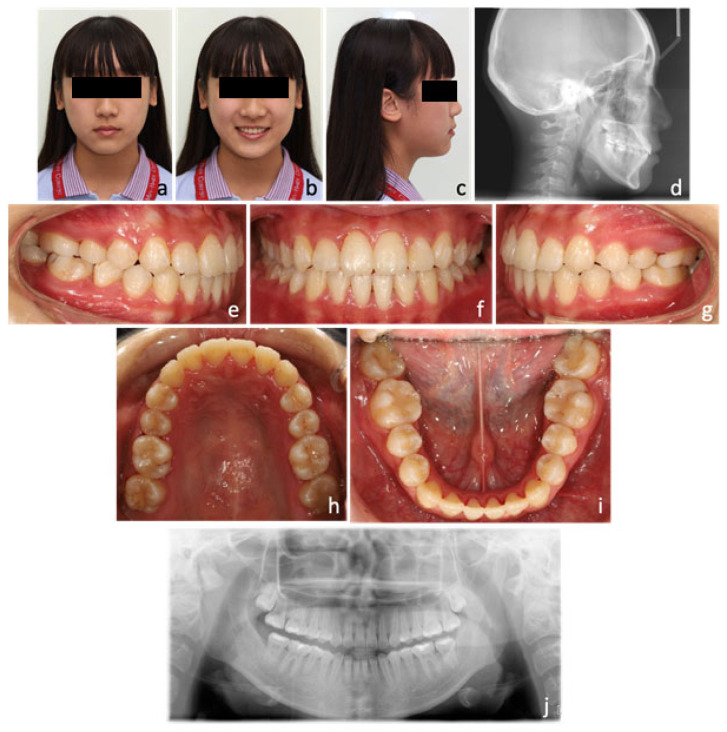
Final photos and radiographs. (**a**,**b**) Extra-oral photos showed more upper incisor display and ideal smile arc. (**c**,**d**) A straight profile was achieved with less retrusive upper lip to the E-line. (**e**–**i**) Intra-oral photos showed the upper narrow arch was expanded and the dental crowding was relieved, anterior and posterior crossbites were corrected and centerlines were coincident. (**j**) The final orthopantographic (OPG) radiograph showed good root parallelism.

After 25 months of treatment, a straight profile was achieved with less retrusive upper lip (upper lip to the E-line: −1.1 mm). More upper incisor display was achieved with an ideal smile arc established (Figure 6a–c).

**Figure 7:**
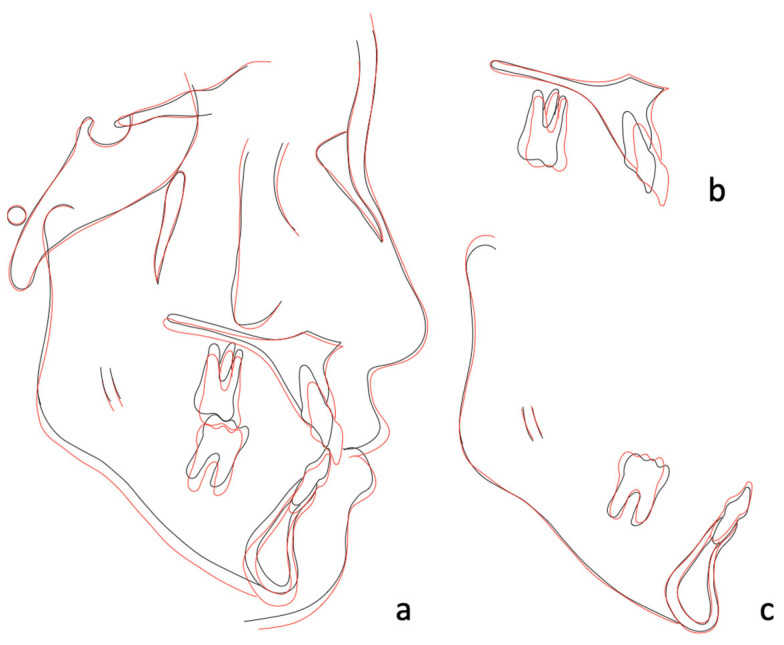
(**a**) Overall superimposition confirmed the profile improvement by maxillary advancement (SNA: 83° to 83.5°) and mandibular backward rotation (SNB: 83° to 82°; SN-MP: 34.5° to 35°), which contributed to the increase in ANB angle (ANB: 0° to 1.5°). (**b**) Maxillary superimposition showed U1 forward movement 4 mm and proclination 7° (U1-SN: 108° to 115°). (**c**) Mandibular superimposition showed L1 extrusion 1.5 mm and proclination 4° (L1-Mn: 87° to 91°); L6 distal tipping 3°.

Cephalometric analysis and superimposition confirmed the profile improvement by maxillary advancement (SNA: 83° to 83.5°) and mandibular backward rotation (SNB: 83° to 82°; SN-MP: 34.5° to 35°). These effects contributed to the increase in ANB angle (ANB: 0° to 1.5°) and facial convexity (G-Sn-Pg’: 7.2° to 8.2°) (Figure 7; Table 2). Upper incisors presented a forward movement of 4 mm, extrusion of 3.5 mm and a proclination of 7° (U1-SN: 108° to 115°). Meanwhile, lower incisors were extruded and proclined by 1.5 mm and 4° (L1-Mn: 87° to 91°) respectively (Figure 7; Table 2). Extrusion of upper and lower incisors contributed to the correction of anterior open bite after MARPE.

Intra-orally, the narrow upper arch was expanded and the dental crowding was relieved. Bilateral class I canine and molar relationships were achieved with good interdigitation. Anterior and posterior crossbites were corrected and centerlines were coincident. Well-aligned dentitions with solid interdigitation and normal overjet/overbite were achieved at the end of treatment (Figure 6e–i).

**Figure 8:**
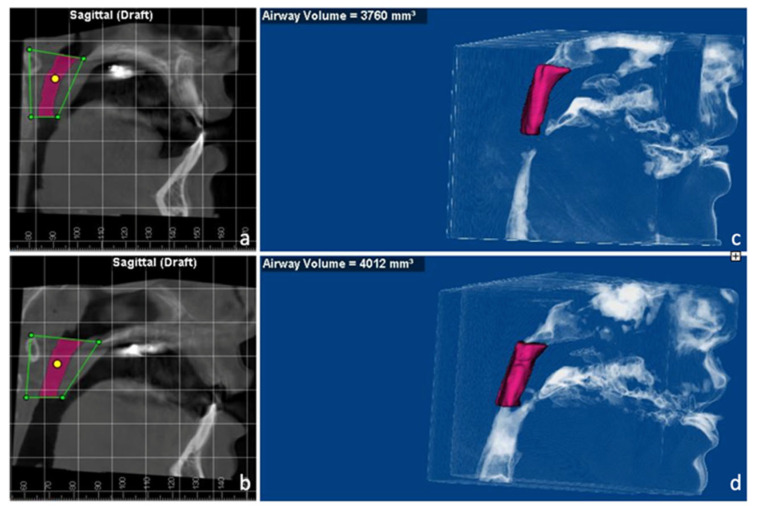
Measurements of the velopharyngeal airway volume. The upper border is defined by a straight line reaching from the PNS and the PPW at the height of the upper limit of the atlas (C1). The lower border of the airway is defined as the end of the soft palate to the posterior pharyngeal wall at the same height. (**a**) Initial cone beam computerized tomography (CBCT) image and borders of velopharyngeal airway. (**b**) Initial velopharyngeal airway volume (3760 mm^3^). (**c**) The post-expansion CBCT image and borders of velopharyngeal airway. (**d**) The post-expansion velopharyngeal airway volume (4012 mm^3^). The volume of velopharyngeal airway was increased from 3760 mm^3^ to 4012 mm^3^ after the MARPE treatment.

The volume of the velopharyngeal airway was measured on CBCT. The upper border was defined by a straight line reaching from the posterior nasal spine (PNS) and the posterior pharyngeal wall (PPW) at the height of the upper limit of the atlas (C1). The lower border of the airway was defined as the end of the soft palate to the PPW at the same height [25] (Figure 8a,b). The volume of the velopharyngeal airway was increased from 3760 mm^3^ to 4012 mm^3^ after MARPE. (Figure 8c,d). The finding is coincident with some airway studies applying MARPE [26,27].

## 3. Discussion

**Figure 9:**
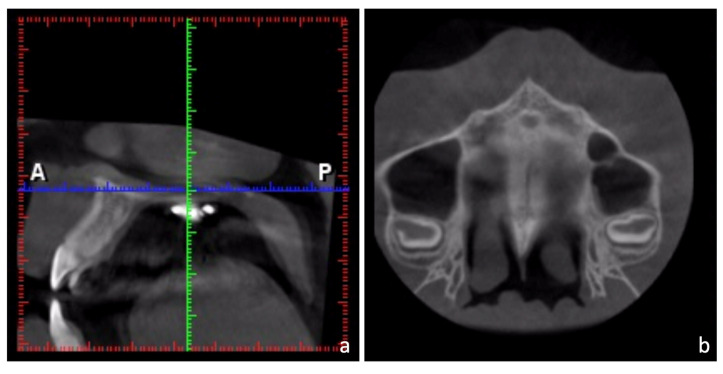
Examination of the maturation stage of the midpalatal suture on pre-expansion CBCT images by applying the method of Angelieri et al. [28] (**a**) Head orientation was done on the CBCT images. (**b**) The axial plane was set in coincidence with the palatal plane and the maturation stage of the midpalatal suture was in stage C.

Maxillary transverse deficiency is usually accompanied by anterior and/or posterior crossbite. According to a recent literature review, maxillary expansion with conventional RPE devices in late adolescence or adulthood may cause adverse side effects including limited skeletal effect, undesirable tooth movement, root resorption, and a lack of long-term stability [6]. Understanding the maturation stage of the midpalatal suture is important to determine which patient can have RPE alone as a less-invasive alternative. Angelieri et al. [28] have presented a novel classification method by using CBCT for assessment of midpalatal suture morphology individually. To ensure successful expansion, we examined the maturation stage of the midpalatal suture of this patient before treatment and found it was in stage C (Figure 9a,b), which implies many initial ossifications along the midpalatal suture have occurred and the start of fusion could be imminent. More force could be required to open the midpalatal suture with increased degree of interdigitation [20]. Considering the maturation stage of the midpalatal suture and the adjacent sutures, treatment with a conventional RPE could be less successful and may lead to unwanted side effects.

Surgical-assisted rapid maxillary expansion (SARPE) contributes to more orthopedic effects with the risk of complex treatment processes, including hemorrhage, injury to the branches of the maxillary nerve, pain, sinus infection, alar base flaring, and relapse [7]. In recent years, microimplant-assisted rapid palatal expansion (MARPE) has shown a significant skeletal effect on expansion. This may also avoid side effects during expansion and the need for additional surgery [9,12].

In this case, anterior crossbite spontaneously improved after MARPE. The main effects resulted from the forward displacement of the maxilla (SNA: 83° to 84°) and backward rotation of the mandible (SN-MP: 34.5° to 35.5°). This result was similar to previous reports by Liao et al., Song et al., and Yılmaz et al. [13,14,15]. The MARPE may not only correct the transverse deficiency but also provide some changes in anteroposterior and vertical dimensions. Backward rotation of the mandible results in a slight increase in anterior total face height [29]. It can make the borderline cases less severe and expand the scope of orthodontic treatment of class III malocclusion.

Facial bones and the adjacent structures are affected by the mechanical forces generated by RPE in adolescent subjects [30]. Cantarella et al. [31] found that the pyramidal process of the palatine bone was pushed out of the pterygoid notch of the pterygoid process, which implied MARPE may disengage the pterygopalatine suture in its lower part. In our case, we found a similar result of disengagement of the pyramidal process from the pterygoid notch in the left pterygopalatine suture on an axial section of post-expansion CBCT. The opening is present between the pyramidal process and the lateral pterygoid plate. (Figure 2b) The disarticulation of the pyramidal process and the pterygoid notch explains the forward movement of the maxilla, which is frequently seen in RPE or MARPE patients. In addition, disarticulation of the pterygopalatine suture and parallel opening of the midpalatal suture result in parallel separation of maxillary halves, and the fulcrum of maxillary rotation is near the proximal portion of the zygomatic process of the temporal bone [31]. This will cause the zygomaticomaxillary complex to be displaced in a lateral direction and rotated outwards around the fulcrum.

Despite the numerous advantages of MARPE, there are still some undesirable adverse effects and complications. Tsai et al. [32] reported the relevant adverse effects including epistaxis, inflammation and swelling of palatal mucosa, difficulty in cleaning, soft tissue impingement, micro-implants loosening, tinnitus, distortion of the expander, failure of suture-opening and asymmetrical expansion. Among these, inflammation and palatal mucosa swelling were the most common complications during MARPE. If inflammation persisted and purulence was noted over the palatal mucosa, a higher dose of Amoxicillin (500 mg every 8 h for 5 days) and the use of chlorhexidine were suggested. All potential adverse effects should be warned in advance, and oral hygiene should be emphasized during treatment. In this case, a questionnaire was given to record the experiences throughout the MARPE procedure, and only “mild pain” was reported with a maximum pain score of 1 (Table 3). The main relevant adverse effect was soft tissue irritation around the expansion device.

After MARPE and initial alignment, the use of TADs over mandibular buccal shelves was effective in providing anchorage for molar distalization to relieve crowding. Chen et al. [22] investigated the spatial limits during mandibular arch distalization, and found both ridge width and available distalization distance were the factors that limited the distance of mandibular distalization. Kim et al. [23] reported that the lingual cortex of the mandibular body was the posterior limit of molar distalization, and CBCT images may provide a better prediction. To ensure predictable tooth movement, we examined available space behind second molars at the beginning and found the distal roots of second molars were close to the lingual cortex of the alveolar process (Figure 2a). Therefore, a large amount of lower arch distalization cannot be anticipated. Only slight distal tipping of molars with TADs was achieved in this case for mild crowding relief.

Elshebiny et al. [33] investigated suitable sites for orthodontic miniscrew insertion over the mandibular buccal shelf in a CBCT study. In consideration of four variables, including cortical bone thickness, bone width, insertion depth, and proximity to nerves, they concluded the level of the distobuccal cusp of the mandibular second molar is the most ideal site for miniscrew insertion. Nucera et al. [34] also found a similar result that the buccal bone corresponding to the distal root of second molar, with screw insertion 4 mm buccal to the cementoenamel junction, could be a suitable site for miniscrew insertion over the buccal shelf.

Nonetheless, the stability of miniscrews is affected by various factors including age, craniofacial skeletal pattern, loading protocol, and anatomic factors of the insertion site [35]. Both hard and soft tissues should be considered when choosing a proper site for miniscrew placement. Better quality and quantity of cortical bone can provide better primary stability, and thin soft tissue is more advantageous because the likelihood of inflammation can be lower [36]. Although previous studies suggested placing miniscrews over the distobuccal cusp level of the mandibular second molar to get more osseous contact, we must be aware of the greatest proximity to the inferior alveolar nerve at this site also. Taking all into consideration, in this case, we placed TADs over mandibular buccal shelves on both sides between lower first and second molars.

Hur et al. [37] investigated the treatment effects of MARPE on an adult patient with obstructive sleep apnea syndrome, and they found that cross-sectional areas of anterior part of the nasal cavity and the upper half of the pharynx were both increased significantly. In a recent literature review, some airway studies have demonstrated that MARPE could offer assistance in respiratory function by enlarging volume and decreasing the total resistance in the upper airway [6]. However, the follow-up period was short in most of the studies. In this case, she did not complain of breathing problems or sleep disturbance from the beginning, so conducting polysomnography (PSG) was not indicated. Post-expansion CBCT images revealed an increase in velopharyngeal volume from 3760 mm^3^ to 4012 mm^3^ though, which may have a positive impact on the volume.

## 4. Conclusions

The present case report demonstrates the successful treatment of a late adolescent patient with class III malocclusion and narrow maxilla. The MARPE with MSE device not only engenders significant transverse correction but also aids in anteroposterior change. Maxillary advancement and mandibular backward rotation are the keys to correct the skeletal class III pattern and lead to a more pleasant profile. MARPE procedure can be an alternative to conventional RPE or SARPE, which are either less effective or more traumatic, in late adolescence or young adults with maxillary transverse deficiency.

## Figures and Tables

**Table 1:** Maxillomandibular transverse discrepancy was 35.5 mm measured from Ricketts Rocky Mountain analysis. Maxillomandibular transverse differential index was 17.5 mm, which was defined by the difference between actual transverse difference and expected transverse difference. When a patient’s index is larger than 5 mm, orthopedic correction, such as RPE, SARPE, or orthognathic surgery should be considered for the transverse correction.

JR-JL	82.2 mm
AG-GA	117.7 mm
Maxillomandibular transverse discrepancy	35.5 mm
Expected transverse difference (14 y/o)	18 mm
Maxillomandibular transverse differential index	17.5 mm

**Table 2:** Initial, post-expansion, and final cephalometric analysis.

	Norm.	Initial	Post-Expansion	Final
Skeletal analysis				
SNA	82+/−2°	83	84	83.5
SNB	80+/−2°	83	82	82
ANB	2+/−2°	0	2	1.5
SN-MP (Go-Gn)	32+/−5°	34.5	35.5	35
Frankfort-mandibular angle (FMA)	25+/−5°	29.8	32	31
Dental analysis				
U1-NA (mm)	3.18~7.34 mm	5.8	6.6	6.1
U1-SN	102.23~115.13°	108	112	115
L1-NB (mm)	3.18~7.34 mm	5.8	5.5	6.1
L1-MP (Go-Gn)	90.56~103.12°	87	87	91
Facial analysis				
E-line (U)	−2.93~0.41 mm	−3.5	−1.6	−1.1
E-line (L)	−1.86~2.1 mm	0	0	0
Facial convexity (G-Sn-Pg’)	4.34~15.84°	7.2	10.5	8.2

**Table 3:** Questionnaire used in this case to record the experiences throughout the MARPE procedure.

Questionnaire Related to Patient’s Experiences during Microimplant-Assisted Rapid Palatal Expansion (MARPE)		
Yes/No Questions:	Yes	No
1. Swelling or inflammation of palatal gingiva	■	□
2. Soft tissue impingement while expansion	■	□
3. Difficult in cleaning around device	□	■
4. Distortions of the device	□	■
5. Microimplant loosening	□	■
6. Epistaxis	□	■
7. Sinusitis	□	■
8. Failure of mid-palatal suture opening	□	■
Open-ended question:		
Any other problem encountered during treatment	□	■
Maximum pain score using numerical rating scale (NRS): 1		

## Data Availability

Not applicable.
